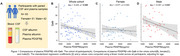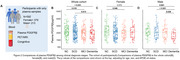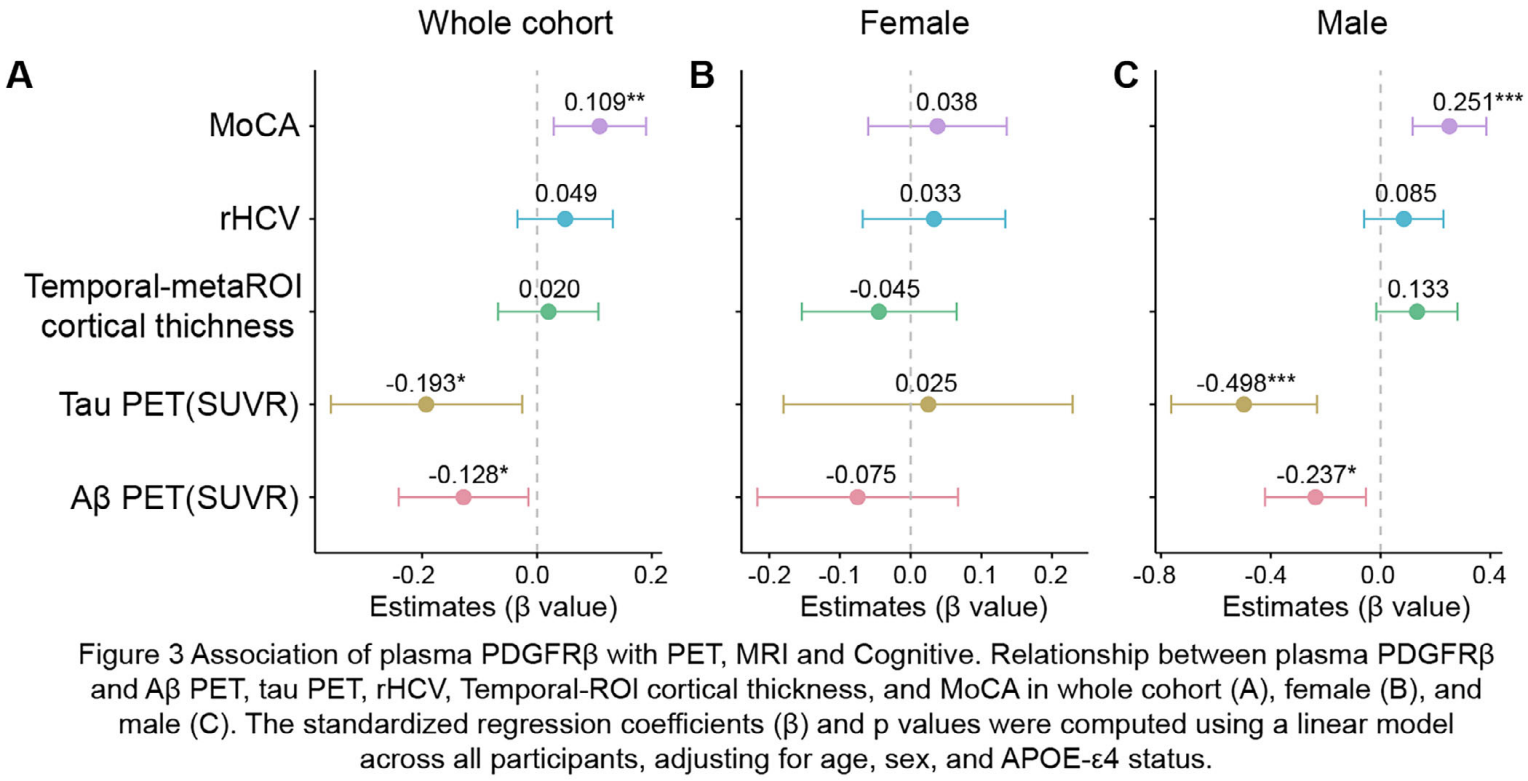# Decreased plasma platelet‐derived growth factor receptor‐β correlates with β‐amyloid, tau, and cognitive decline in male Alzheimer's disease

**DOI:** 10.1002/alz70856_101847

**Published:** 2025-12-25

**Authors:** Jieyin li, Anqi Li, Laihong Zhang, Guoyu Lan, Jie Yang, Zhen Liu, Shaohua Ma, Xuhui Chen, Dai Shi, Tengfei Guo

**Affiliations:** ^1^ Institute of Neurological and Psychiatric Disorders, Shenzhen Bay Laboratory, Shenzhen, Guangdong, China; ^2^ Shenzhen Bay Laboratory, Shenzhen, Guangdong, China; ^3^ Tsinghua Shenzhen International Graduate School (SIGS), Tsinghua University, Shenzhen, China; ^4^ Peking University Shenzhen Hospital, Shenzhen, Guangdong, China; ^5^ The Seventh Affiliated Hospital, Sun Yat‐sen University, Shenzhen, Guangdong, China; ^6^ Institute of Biomedical Engineering, Shenzhen Bay Laboratory, Shenzhen, China; ^7^ Peking University Shenzhen Graduate School, Shenzhen, Guangdong, China

## Abstract

**Background:**

Cerebrospinal fluid (CSF) platelet‐derived growth factor receptor‐β (PDGFRβ) concentration, as well as CSF/plasma albumin ratio (Qalb), have been regarded as the biomarkers reflecting blood‐brain barrier (BBB) damage. However, it is still unclear whether plasma PDGFRβ levels relate to BBB damage as well as how it correlates with Alzheimer's disease (AD).

**Method:**

We tested Qalb and plasma PDGFRβ concentrations among 93 participants with concurrent CSF and plasma samples from the Greater‐Bay‐Area Healthy Aging Brain Study (GHABS) Chinese aging cohort. We investigated the association between Qalb and plasma PDGFRβ in the whole cohort, females and males. Additionally, we measured plasma PDGFRβ in 592 GHABS participants who only had plasma samples, including 122 normal control (NC), 277 subjective cognitive decline (SCD), 110 mild cognitive impairment (MCI), and 83 dementia. Among 592 participants, 277, 143, 469, and 542 had Aβ PET, tau PET, structural MRI, and MoCA scores, respectively. We investigated the association of plasma PDGFRβ with Aβ‐PET, tau‐PET, residual hippocampal volume (rHCV), temporal‐MeraROI cortical thickness, and MoCA.

**Result:**

Plasma PDGFRβ concentrations were negatively associated with Qalb in the whole cohort (standardized β (β_std_) = ‐0.225 [95% confidence interval (ci), ‐0.426 ‐ ‐0.025], *p* =  0.028) and males (β_std_ = ‐0.347 [95% ci, ‐0.641 ‐ ‐0.053], *p* =  0.021) but not in females (Figure 1). MCI and dementia patients showed lower plasma PDGFRβ than NC individuals in the whole cohort and males (Figure 2). Additionally, lower plasma PDGFRβ levels were related to higher Aβ PET and tau PET and lower MoCA scores in the whole cohort and males (Figure 3).

**Conclusion:**

These findings demonstrated that decreased plasma PDGFRβ levels are related to BBB damage, providing a potential plasma biomarker to detect and monitor BBB leakage in AD, particularly in males.